# Use of human amniotic epithelial cells in mouse models of bleomycin-induced lung fibrosis: A systematic review and meta-analysis

**DOI:** 10.1371/journal.pone.0197658

**Published:** 2018-05-17

**Authors:** Fang He, Aiting Zhou, Shuo Feng

**Affiliations:** 1 Key Laboratory of Cell Engineering of Guizhou Province, The Affiliated Hospital of Zunyi Medical College, Zunyi, Guizhou, China; 2 Department of Spine Surgery, The Affiliated Hospital of Zunyi Medical College, Zunyi, Guizhou, China; 3 Beijing Hospital of Traditional Chinese Medicine, Capital Medical University, Beijing Institute of Traditional Chinese Medicine, Beijing, China; Centre National de la Recherche Scientifique, FRANCE

## Abstract

**Background:**

Idiopathic pulmonary fibrosis (IPF) urgently requires effective treatment. Bleomycin-induced lung injury models are characterized by initial inflammation and secondary fibrosis, consistent with the pathological features of IPF. Human amniotic epithelial cells (hAECs) exhibit good differentiation potential and paracrine activity and are thus ideal for cell-based clinical therapies. The therapeutic effects of hAECs on lung fibrosis are attributed to many factors. We performed a systematic review of preclinical studies investigating the treatment of pulmonary fibrosis with hAECs to provide suggestions for their clinical use.

**Methods:**

PubMed and EMBASE were searched for original studies describing hAEC therapy in animal bleomycin-induced pulmonary fibrosis models. After quality assessments, the number and species of experimental animals, bleomycin dose, hAEC source and dosage, time and route of administration of transplanted cells in animals, and time animals were euthanized in nine controlled preclinical studies were summarized. Ashcroft scores, lung collagen contents, inflammatory cells and cytokines were quantitatively and/or qualitatively analyzed in this review. Publication bias was also assessed.

**Results:**

Each of the nine preclinical studies have unique characteristics regarding hAEC use. Ashcroft scores and lung collagen contents were decreased following hAEC transplantation in bleomycin-injured mice. Histopathology was also improved in most studies following treatment with hAECs. hAECs modulated macrophages, neutrophils, T cells, dendritic cells and the mRNA or protein levels of cytokines associated with inflammatory reactions (tumor necrosis factor-α, transforming growth factor-β, interferon-γ and interleukin) in lung tissues of bleomycin-injured mice.

**Conclusions:**

hAECs alleviate and reverse the progression of bleomycin-induced lung fibrosis in mice and may represent a new clinical treatment for IPF. hAECs exert anti-inflammatory and anti-fibrotic effects by modulating macrophage, neutrophil, T cell, dendritic cell and related cytokine levels in mice with bleomycin-induced lung fibrosis. Cell generation and the route, source and timing of hAEC transplantation all determine the therapeutic effectiveness of hAECs.

## Introduction

Lung injury accompanied by inflammation, cell death and inflammatory cytokine production in response to chemical and/or physical stimuli may ultimately result in pulmonary fibrosis. Idiopathic pulmonary fibrosis (IPF) is induced by the abovementioned factors and is characterized by a high mortality rate and diffuse alveolar inflammation and fibrosis, consequently threatening human health [1]. Immunosuppressive drugs are widely applied treatments for IPF, but their curative effects are not satisfactory. Lung transplantation is the only option for patients with end-stage lung disease. The bleomycin-induced model of lung injury is consistent with the developmental process of IPF and is a well-characterized model of the initial inflammation and subsequent fibrosis [2]. These animal models are suitable and convenient for preclinical studies of these diseases. Bone marrow, umbilical cord and amniotic fluid-derived mesenchymal stem cells (MSCs) exert certain curative effects on mouse models of pulmonary fibrosis, and some MSC therapies have entered clinical trials. However, the differentiation capacity, engraftment rate and secretory function of MSCs must be more precisely elucidated [3]. Human amniotic epithelial cells (hAECs) are derived from the amniotic membrane of the placenta after childbirth and retain the earliest characteristics of embryonic stem cells, such as expression of the surface markers Oct-3/4, SSEF-3, SSEA-4, Rex-1 and BMP-4. hAECs differentiate into endodermal, ectodermal and mesodermal lineages, lack telomerase activity, do not pose a tumorigenic risk and uniquely express the epithelial cell marker cytokeratin 19. hAECs are also advantageous because they are retrieved non-invasively from a rich source and exert paracrine functions, similar to MSCs. Most importantly, hAECs differentiate into alveolar epithelial cells both in vitro and in mice in vivo, representing an ideal cell-based clinical therapeutic option for lung regeneration [4,5]. The therapeutic effects of hAECs on pulmonary fibrosis are attributed to many factors, but the underlying mechanisms are not completely understood, directly impacting their clinical applications. Therefore, we analyzed the therapeutic effects of hAECs on animal models of bleomycin-induced fibrosis and summarized the characteristics of preclinical studies utilizing hAECs to treat bleomycin-induced pulmonary fibrosis in mice. Our purpose was to provide an effective reference for the clinical application of hAECs in the treatment of IPF.

## Methods

### Search strategy and selection criteria

A systematic search of relevant articles was performed according to the recommendations of the preferred Reporting Items for Systematic Reviews guidelines [6], which are briefly described in S1 Table. We also deposited our laboratory protocols at protocols.io with the identifier dx.doi.org/10.17504/protocols.io.pjqdkmw. Relevant studies were identified by searching PubMed and EMBASE (through June 2017). MeSH terms combined with free words were used to identify the search terms. Terms used in the search included “Amniotic Epithelial Cells” and “Pulmonary” (refer to S3 Table). We also performed a manual search using the reference lists of key articles published in English. Only English publications were included in the search.

### Study selection and data extraction

The inclusion criteria were: (1) only hAECs were transplanted into the animals, (2) the groups in the experiment included an epithelial cell transplantation group and a saline transplantation control group, and (3) the model of pulmonary fibrosis in the animal experiment was induced with bleomycin.

Studies meeting the following criteria were excluded: (1) the transplanted cells were non-natural (transgenic treatment, inducer induction, etc.), (2) the animals used were treated (knockout of genes, drug treatment, etc.), and (3) the studies were duplicate documents and literature reviews.

Two independent investigators (He and Zhou) reviewed the study titles, abstracts and methods. Ambiguous or controversial articles were assessed by reading the full text. All data were extracted independently by the two reviewers (He and Zhou). Disagreements were resolved by a third reviewer (Feng). The data included the first author’s name, publication date, country of origin, recipient animal species, total number of cases, study design and parameters observed. If the data required for the quantitative analysis were missing or only expressed graphically, we attempted to contact the authors for further information. If a response was not received, we extracted data from graphs using digital ruler software or the study was excluded.

### Risk of bias

The risk of bias in each experiment was assessed by two reviewers (FH and ATZ) using the SYRCLE risk of bias tool. The SYRCLE risk of bias tool is based on the Cochrane Risk of Bias tool and is suitable for animal intervention studies [7]. A third investigator (SF) was recruited to resolve differences of opinion. Ten assessment items related to selection, performance, detection, attrition, reporting and other biases in the SYRCLE tool were assessed and scored as having a low, high, or unclear risk of bias. Responses of “yes”, “no”, or “do not explicitly state” to questions in the tool respectively indicate a “low risk of bias”, “high risk of bias” or “unclear risk of bias”.

### Statistical analysis

Ashcroft scores and lung collagen contents were reported as pooled relative risks and 95% confidence intervals (CIs) and analyzed using Review Manager Version 5.3. Because heterogeneity in both Ashcroft scores and lung collagen contents was reported, a random-effects model was used, and heterogeneity was evaluated using the I^2^ statistic. Due to data complexity, the results obtained from analyses of inflammatory cells and related cytokines are presented descriptively. We extracted key parameters that may affect end therapy, such as the animal species used, the source and passage of the hAECs, the route and number of injected cells and the injection and outcome times after the establishment of the bleomycin-induced lung fibrosis model. We assessed the possibility of publication bias by constructing a funnel plot and performing Egger’s test to determine the effect size of each trial on the standard error.

## Results

### Search results and study characteristics

**Fig 1:**
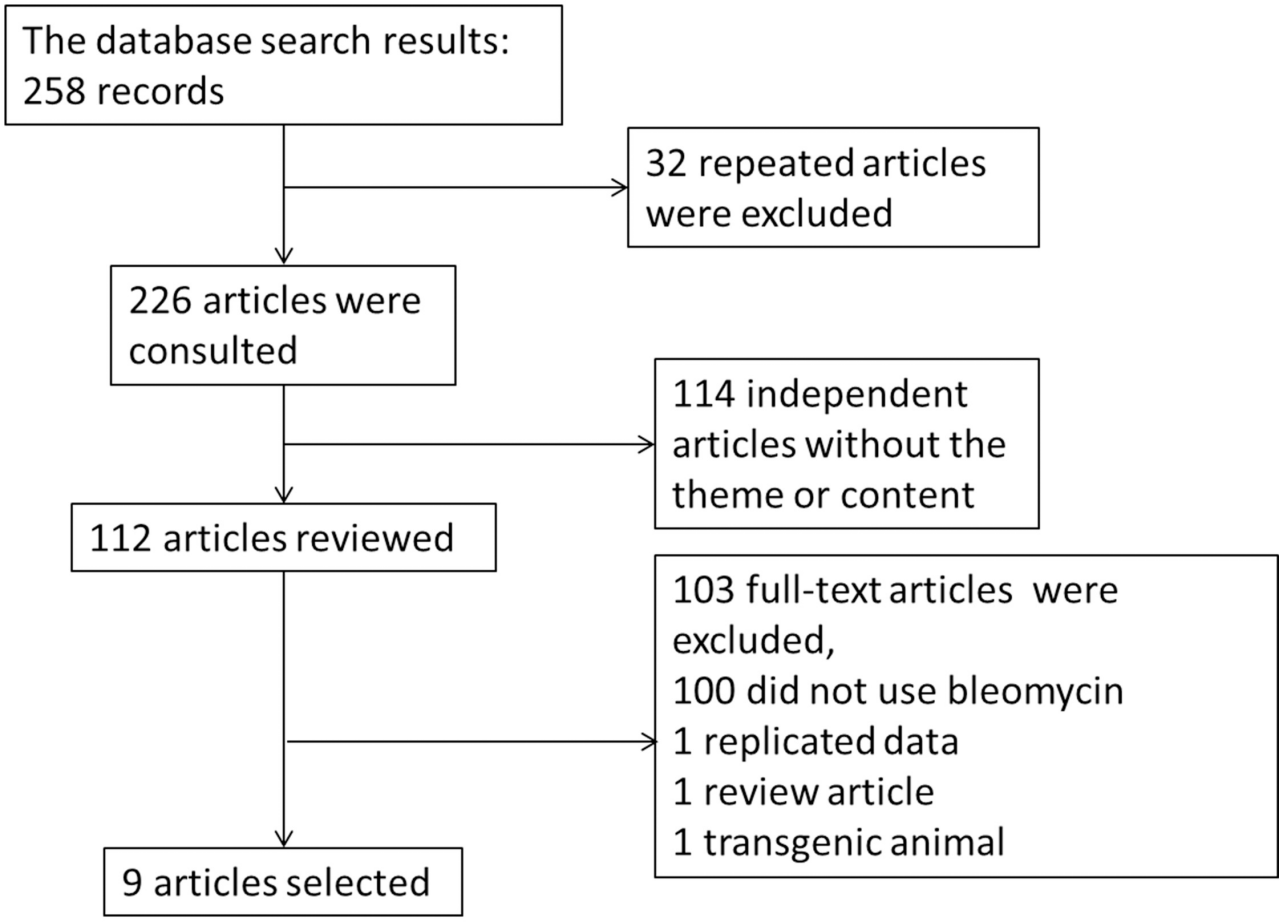
Included and excluded studies.

Our systematic database search yielded 258 records. Following deduplication and screening, 9 articles were included in the review (Fig 1 and Table 1). These studies were published over a six-year period (2010–2016) and corresponded to 17 unique experiments (Table 2). One study did not specify the number of mice used [8]. All studies were performed in Australia, based on the location of the first author. C57BL/6 mice were used in 7 research studies, while SCID mice and wild-type mice were used in the other two studies. hAECs were transplanted between 24 h and 14 d after treatment with bleomycin. Transplanted P0/P5 hAECs were derived from normal-term or preterm pregnancies delivered by Cesarean section. An efficacy test was performed within three weeks after bleomycin treatment. The number of cells administered ranged from 1×10^6^ to 4×10^6^ cells. The routes of transplantation included intravenous (i.v.) and intraperitoneal (i.p.) administration; i.p. injection was the primary method used. The findings generally included histopathological changes, inflammatory infiltration, collagen deposition and cytokine levels in the lung tissue and related molecular targets in saline/hAEC-treated mice with bleomycin-induced injury (Table 1).

**Table 1:** Characteristics of included studies.

First author (year)	N (hAECs/saline)	Animal (age/gender)	Ble Dose (route)	Cell source	Cell Dose (×10^6^)	Time (route)	Cull time
Moodley 2010 [5]	8/8	SCID mice (8 weeks/?)	0.15 mg (i.n.)	hAECs (term)	1	24 h/14 d (i.v.)	2/4 w after Ble
Lim 2013 [9]	8/8	C57/BL6 mice (8 to 10 weeks/female)	0.3 U (i.n.)	hAECs (term/preterm)	4	24 h (i.p.)	7/14 d after Ble
Vosdoganes 2013 [10]	8/8	C57BL/6 mice (6 to 8 weeks/female)	0.03 U/kg (i.n.)	hAECs (term)	4	7/14 d (i.p.)	14 d after Ble
Tan 2014 [11]	6/6	C57BL/6 mice (6 to 8 weeks/female)	0.3 U (i.n.)	hAECs	4	24 h (i.p.)	1/3/5/7 d after Ble
Tan 2017 [12]	19/19	C57BL/6 mice (8 to 12 weeks/female)	15 IU (i.n.)	hAECs (term)	4	24 h (i.p.)	1/3/5/7 d after Ble
Murphy 2012 [13]	23/15	wild-type mice (6 to 8 weeks/female)	7 U/kg (i.n.)	hAECs	4	24 h (i.p.)	7/14 d after Ble
Moodley 2013 [14]	8/8	C57BL/6 mice (8 weeks/female)	0.15 mg (i.n.)*	hAECs (P0/P5)	1	72 h (i.v.)	1/3 w after cell injection
Zhu 2016 [8]	?	C57BL/6 mice (6 to 8 weeks/female)	0.3 IU (i.n.)	hAECs (term/preterm)	4	24 h (i.p.)	14 d after Ble
Murphy 2011 [15]	14/14	C57BL/6 mice (6 weeks/female)	8 U/kg (i.n.)	hAECs	4	24 h (i.p.)	7/14 d after Ble

hAECs: human amniotic epithelial cells

i.v.: intravenous injection

i.p.: intraperitoneal injection

i.n.: intranasal; and

Ble: bleomycin.

*Bleomycin was administered at two time points, with a one-week interval between the first and the second injections.

**Table 2:** Summary of inflammation and fibrosis.

Study	Lung collagen	Ashcroft score	Lung inflammatory cells	α-SMA	Lung TNF-α	Lung TGF-β	Lung IL-6	Lung IFN-γ	Lung IL-1	Lung IL-2	Lung IL-4	Lung IL-10	MIF
Moodley 2010 [5] (culled at 14 d)	↓^a^	↓	↓ (inflammation score)		↓ (mRNA) NS (Pro)	↓ (mRNA, Pro)	↓ (mRNA, Pro)	↓ (mRNA)	↓ (mRNA, Pro)	↓ (mRNA)		↑ (mRNA)	↑ (mRNA)
Moodley 2010 [5] (culled at 28 d)	↓^a^ (i.v. hAECs at 14 d)	↓	↓ (inflammation score)										
Moodley 2013 [14] (P0 hAECs)	NS^b^ (21 d)	NS (21 d)	↓ (7 d, inflammation score and CD45+ cells)		NS (Pro)	↓ (Pro, 21 d)	NS (Pro, 7 d)		NS (Pro)				
Moodley 2013 [14] (P5 hAECs)	NS^b^ (21 d)	NS (21 d)	NS (7 d, inflammation score and CD45+ cells)		NS (Pro)	↓ (Pro, 21 d)	↓ (Pro, 7 d)		NS (Pro)				
Murphy 2011 [15] (culled at 7 d)	NS^c^	↓	↓ (macrophages and MPO+ neutrophils)		↓ (mRNA)	↓ (mRNA)	↓ (mRNA)	↓ (mRNA)					
Murphy 2011 [15] (culled at 14 d)	↓^c^	↓		↓			↓(mRNA)						
Murphy 2012 [13]	↓^c^ (14 d)	↓ (14 d)	↓ (7 d, macrophages and MPO+ neutrophils)	↓ (14 d)		↓ (mRNA,7 d)	↓ (mRNA, 7 d)		↓ (mRNA, 7 d)				↓ (mRNA, 7 d)
Tan 2017 [12] (culled at 3 d)			NS (T cells), NS (macrophages), NS (CD11b+ neutrophils), NS (dendritic cells)		NS (Pro)		NS (Pro)			NS (Pro)	↑ (Pro)	NS (Pro)	
Tan 2017 [12] (culled at 5 d)			NS (T cells), ↓ (macrophages), NS (CD11b+ neutrophils), ↓ (dendritic cells)		NS (Pro)		NS (Pro)			NS (Pro)	NS (Pro)	↓ (Pro)	
Tan 2017 [12] (culled at 7 d)			↓ (T cells), ↓ (macrophages), NS (CD11b+ neutrophils), NS (dendritic cells)		NS (Pro)		↓ (Pro)			↓ (Pro)	NS (Pro)	NS (Pro)	
Vosdoganes 2013 [10] (I.P. hAECs at 7 d, culled at 14 d)	NS^d^		↑(CD45 density)	NS		↓ (mRNA)							
Vosdoganes 2013 [10] (I.P. hAECs at 14 d, culled at 21 d)	↓^d^		↓ (CD45 density)	↓		↓ (mRNA)							
Lim 2013 [9] (term hAECs)	↓^d^ (14 d)	↓(14 d)	↓ (? d, macrophages)	↓ (? d)									
Lim 2013 [9] (preterm hAECs)	↓^d^ (14 d)	NS (14 d)	NS (? d, macrophages)	↓ (? d)									
Zhu 2016 [8] (term hAECs)	↓^e^ (14 d)												
Zhu 2016 [8] (preterm hAECs)	NS^e^ (14 d)												
Tan 2014 [11]			↓ (7 d, macrophages)										

^a^% collagen/mg in lung tissue

^b^measured by determining hydroxyproline levels

^c^measured using Sirius red staining

^d^measured by determining the collagen area

^e^not quantitated; ↑: increased compared with the saline group (<0.05); ↓: decreased compared with the saline group (<0.05); NS: not significant; Pro: protein; d: day; i.v.: intravenous injection; i.p.: intraperitoneal injection; hAECs: human amniotic epithelial cells; and MPO: myeloperoxidase.

### Risk of bias

The risk of bias was assessed in 9 articles using the SYRCLE risk of bias tool (S2 Table). None of the studies were determined to have a low risk of bias. Baseline data were similarly reported for the hAEC group and saline group in all studies. None of the studies clearly described the method of random sequence generation, allocation concealment, the random housing of animals, and the blinding of caregivers and/or examiners. All studies were scored as having a low risk of bias in the blinding of outcome assessors and addressing incomplete outcome data and were free from selective outcome reporting categories. Five studies did not describe the random selection method for outcome assessment. Four studies reported that random animal selection was used to assess experimental results. Other problems that lead to a high risk of bias, such as pharmaceutical pollution, conflicts of interest, and wrong units, were not observed in any study.

### Ashcroft score

**Fig 2:**
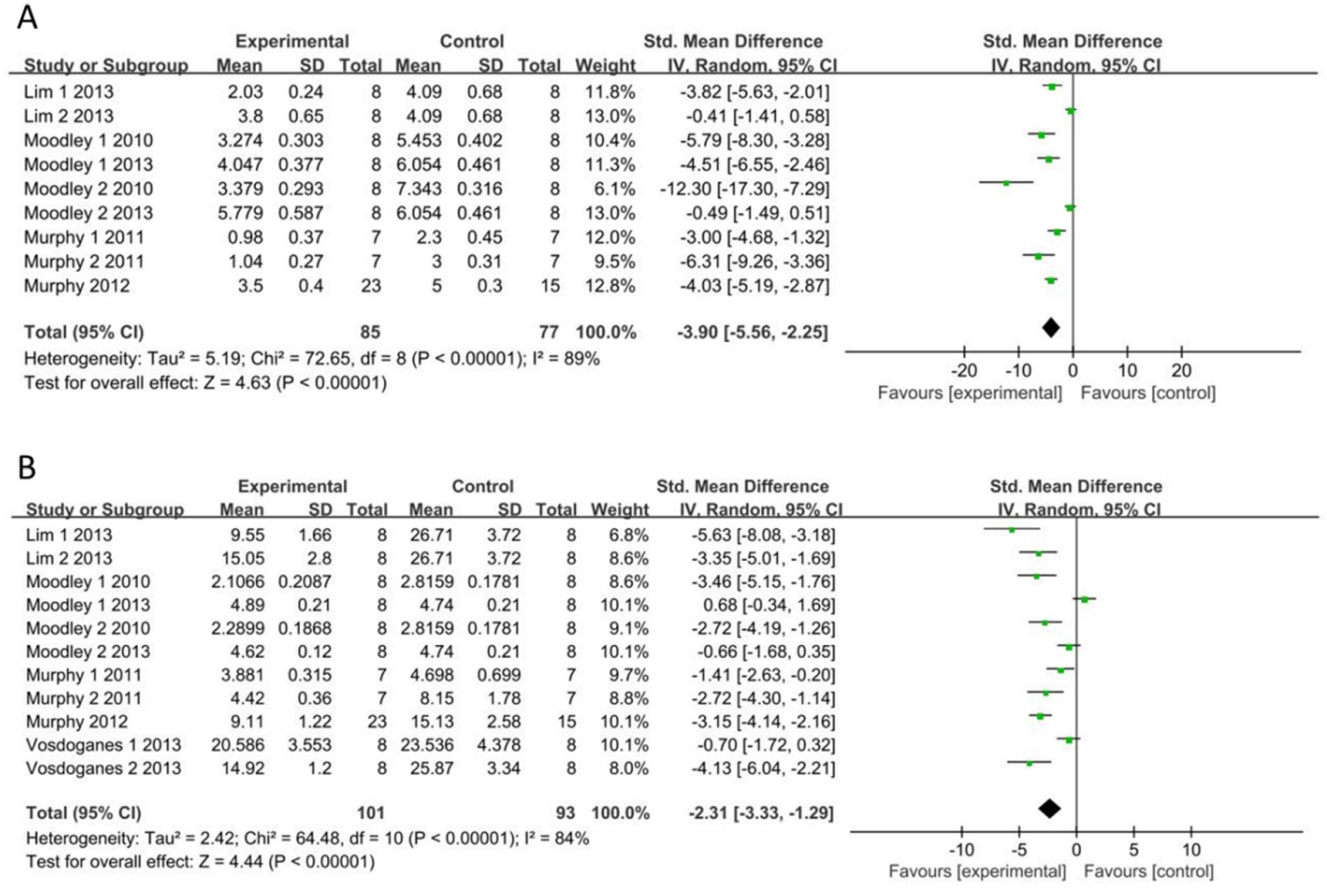
**Forest Plots of Ashcroft Scores (A) and Lung Collagen Contents (B).** CI: confidence interval; IV: independent variable; SD: standard deviation; Experimental: hAEC administration group; and Control: saline control group.

**Fig 3:**
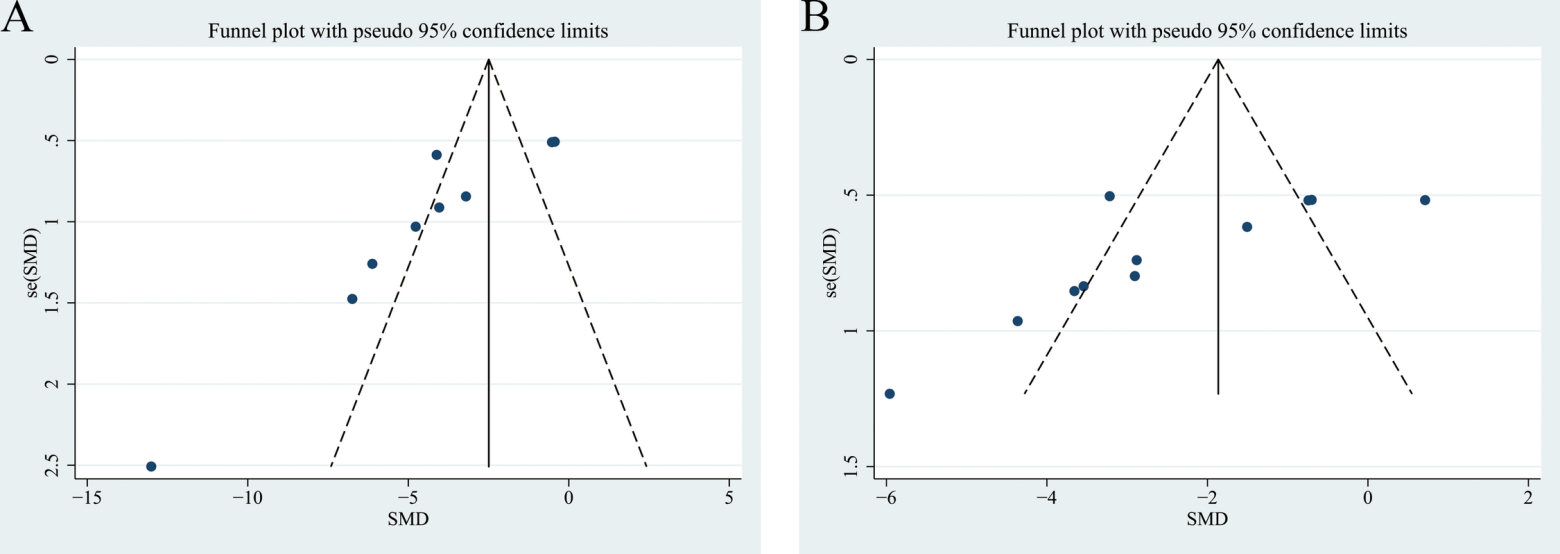
**Funnel Plots of Ashcroft Scores (A) and Lung Collagen Contents (B).** SE: standard error; and SMD: standard mean difference.

**Fig 4:**
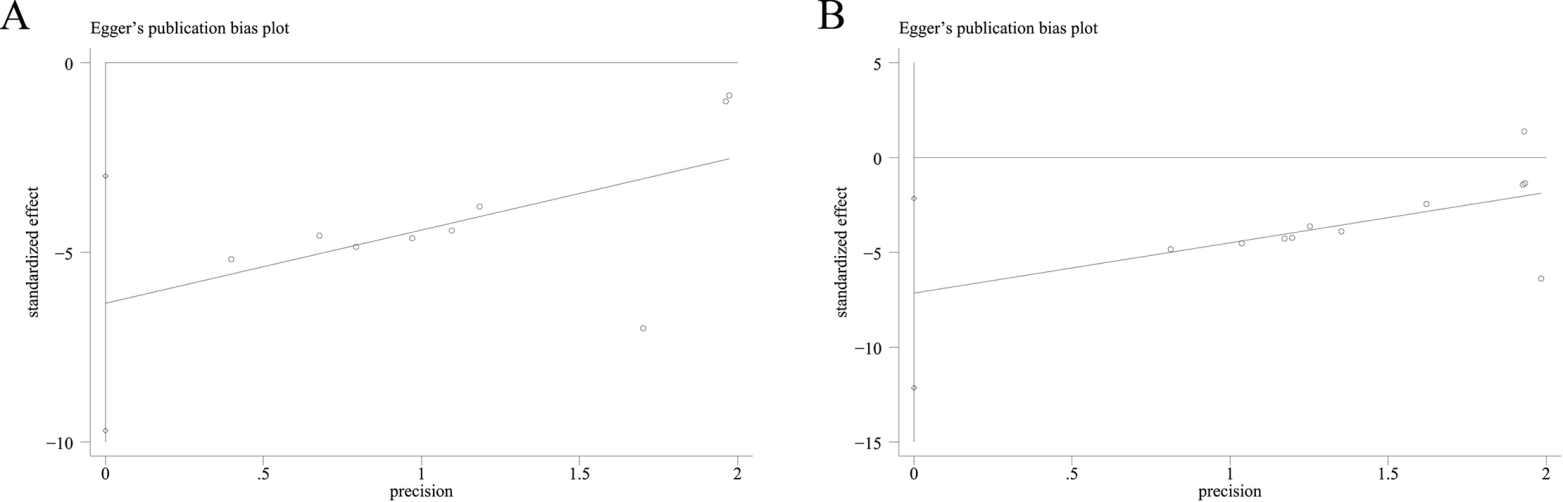
Egger’s Plots of Ashcroft Scores (A) and Lung Collagen Contents (B).

The Ashcroft score was measured in 9 independent studies [5,9,13–15]. Ashcroft scores of the saline and hAEC groups were analyzed as continuous variables using the means and standard deviations. A pooled analysis of the nine studies revealed a 3.90-fold higher Ashcroft score in the saline control group than in the hAEC experimental group (standard mean difference, -3.90; 95% CI, -5.56, -2.25; P<0.05; (Fig 2A)). A funnel plot of the Ashcroft scores of the saline and hAEC groups revealed that their values were not distributed around the overall estimate (Fig 3A). Egger’s plot of the Ashcroft scores revealed existing potential publication bias (Fig 4A) and Egger’s P value was 0.003.

### Lung collagen content

Lung collagen contents were assessed in 13 independent studies, but two studies did not report these data or include charts for quantitative analysis [5,8–10,13–15]. Therefore, the data from 11 studies related to collagen content were extracted from 9 articles. Collagen, collagen area, Sirius red staining and hydroxyproline levels were quantitatively analyzed to directly or indirectly assess collagen volume. According to the results, a significantly higher collagen content was observed in the saline control group than in the hAEC experimental group (standard mean difference, -2.31; 95% CI, -3.33, -1.29; P<0.05; Fig 2B). A funnel plot of the collagen contents in the saline and hAEC groups revealed that their values were not distributed around the overall estimate (Fig 3B). Egger’s plot of the collagen contents revealed existing potential publication bias (Fig 4B) and the Egger’s P value was 0.01.

### Alpha-smooth muscle actin (α-SMA)

α-SMA is a protein marker of myofibroblasts. α-SMA levels were assessed in four articles [9,10,13,15]. α-SMA levels in mice were decreased by hAEC administration 7 d after bleomycin treatment, according to Vosdoganes et al. [10] (Table 2), but this decrease was not significant.

### Inflammatory cells

Inflammatory cells were evaluated in all articles, except for the study by Zhu et al. [8] In the study by Moodley et al. [5,14], inflammatory scores decreased after hAEC administration, but not with P5 hAECs, and changes in the number of CD45+ cells were consistent with inflammatory scores. According to Vosdoganes et al. [10], the numbers of CD45+ cells increased and decreased at different time points. The number of macrophages decreased following hAEC treatment at 5 or 7 d after bleomycin injury [11–13,15], but this number did not decrease following the administration of preterm hAECs at 3 d after injury [9,12]. Neutrophil numbers were decreased at 7 d in the study by Murphy et al. [13,15], but the number of these cells was not significantly different in the study by Tan et al. [12]. T cell numbers decreased following hAEC injection at 7 d but not at 3 or 5 d, while dendritic cell numbers decreased at 5 d but not at 3 or 7 d after injury [12] (Table 2).

### Cytokines

Lung tumor necrosis factor α (TNF-α) levels were measured in seven studies [5,12,14,15]. TNF-α mRNA levels decreased following hAEC administration in two studies, while TNF-α protein levels did not exhibit significant differences between the saline and hAEC groups in any of the studies examined. Transform growth factor-β (TGF-β) expression was assessed in seven studies [5,10,13,14,15], and levels of the TGF-β mRNA and protein decreased upon hAEC administration in all studies. IL-6 levels were measured in nine studies [5,12–15]. Levels of the IL-6 mRNA decreased following hAEC application in the studies by Murphy et al. [13,15] and Moodley et al. [5,14]. Levels of the IL-6 protein decreased following hAEC administration in three studies, but three other studies did not report a significant difference following hAEC administration. Levels of the interferon-γ (IFN-γ), IL-1, IL-2, IL-4, IL-10, and MIF mRNAs and proteins increased and decreased under different conditions [12]. IFN-γ levels were measured in two studies [5,15], and levels of the IFN-γ mRNA decreased following hAEC administration at 7 and 14 d after injury. IL-1 expression was measured in four studies [5,13,14]. Levels of the IL-1 mRNA decreased following hAEC administration at 7 and 14 d after injury in the studies by Moodley et al. and Murphy et al., while levels of the IL-1 protein decreased following hAEC administration at 14 d after injury, according to Moodley et al., but were not significantly different at 7 d, according to two other studies. Levels of the IL-2 mRNA decreased following hAEC administration at 14 d [5], and levels of the IL-2 protein decreased at 7 d, but not at 3 or 5 d, after injury [12]. Levels of the IL-4 protein increased after hAEC administration at 3 d but not at 5 or 7 d after injury [12]. The expression of the IL-10 mRNA decreased following hAECs administration at 14 d after injury [5]. Levels of the IL-10 protein decreased in mice injected with hAECs at 5 d but not at 3 or 7 d after injury [12]. Finally, the expression of the MIF mRNA decreased at 7 d and increased at 14 d after injury following hAEC treatment [5,13] (Table 2).

## Discussion

This study evaluated various measures of the therapeutic effects of hAECs on bleomycin-induced pulmonary fibrosis in mice described in 9 articles. Major indices of pulmonary fibrosis, such as the Ashcroft score and collagen content, were analyzed quantitatively. The mRNA or protein expression levels of related cytokines were summarized. The anti-inflammatory and anti-fibrotic effects of hAECs and related mechanisms were further investigated.

### Treatment effects

The Ashcroft score is an index that is widely used to evaluate pulmonary fibrosis [16]. The lung collagen content directly reflects the effects of early inflammatory infiltration and the extent of pulmonary fibrotic lesions, which are important pathological indicators of lung function. The Ashcroft score, which represents the extent of fibrosis, the collagen content and the α-SMA content, provides an indication of the degree of pulmonary fibrosis. Therefore, we collected the above indices from 9 articles and quantitatively assessed the effects of hAECs on pulmonary fibrosis. The Ashcroft scores and lung collagen contents in mice in the hAEC experimental groups were more indicative of improvements than the corresponding values of the mice in the saline control groups, indicating that hAECs prevent bleomycin-induced pulmonary fibrosis.

### Risk of bias

The lack of studies in the experimental report was indicated by the SYRCLE risk of bias tool. None of the 9 studies included in the present analysis was assessed as having a low risk of bias, based on the reporting entries included in the tool. In all domains, only the details of the scientific method that were specifically stated were scored as having a low risk of bias. Therefore, studies may have utilized these methods in their experiment but did not explicitly describe the methods in the article. Our review emphasizes this widespread shortcoming and suggests that a higher reporting standard is required in publishing. Moreover, improvements in the description of experimental details, particularly in preclinical studies of hAEC therapy for bleomycin-injured mice, may be helpful for subsequent clinical experiments on hAEC therapy for IPF. We recommend the use of a checklist such as the SYRCLE risk of bias tool to design future preclinical studies and reduce internal bias.

### Inflammatory cell responses

Large numbers of eosinophils, T cells, neutrophils and dendritic cells are present in the bronchoalveolar lavage fluid of patients with IPF [17,18]. These cells also play important roles in the development of bleomycin-induced lung fibrosis in mice.

Macrophages exhibit phagocytosis, trophic, regulatory and repair functions [19]. Bleomycin-induced pulmonary fibrosis increases lung IL-6 and TNF-α levels and subsequently elevates MCP-1 levels; MCP-1 then recruits macrophages to the lung tissue [15]. hAEC administration significantly reduced IL-6 and MIP gene expression in wild-type mice, but this effect was not observed in surfactant protein C knockout mice that are susceptible to lung injury due to impaired macrophage function. Based on these data, macrophage regulation in the host is essential for hAEC-mediated repair of lung injury [13]. hAECs reduce the total number of macrophages in the lung tissue, enhance the phagocytic activity of macrophages, and promote the M2 polarization of macrophages by Tregs to exert anti-inflammatory effects on mice [11]. hAECs also regulate the balance between Th1 and Th2 cells mediated by related cytokines, thus causing T cells to exert their anti-inflammatory and anti-fibrotic effects [10].

Neutrophils contribute to airway damage by releasing proteases and oxygen species, leading to the loss of alveoli, increased mucus production, and mucociliary dysfunction [20]. Lipoxin A4 is a lipid mediator of the anti-inflammatory pathway that regulates neutrophil infiltration, macrophage polarization and apoptotic polymorphonuclear (PMN) neutrophils [12]. LXA4 and aspirin-triggered lipoxin analogs improve alveolarization in a neonatal model of hyperoxia-induced injury and prevent inflammation and fibrosis in bleomycin-induced lung injury [21–23]. hAECs reduce the myeloperoxidase (MPO) activity in lipoxin A4-stimulated neutrophils. Moreover, Tan et al. [12] did not observe differences in the numbers of CD11b+ neutrophils between injured and hAEC-treated mice at any time point examined, suggesting that hAECs do not alter neutrophil infiltration at the site of injury. The authors hypothesized that hAECs alleviate lung damage by modulating the activity of neutrophils. According to Murphy et al., the administration of hAECs reduced the number of MPO+ neutrophils. The inconsistencies between the results reported by Tan et al. and Murphy et al. may be attributed to the different criteria employed to identify neutrophils. hAECs clearly reduce MPO expression in neutrophils, resulting in decreased activity and activated apoptosis of PMNs.

Dendritic cells are antigen-presenting cells that influence T cell proliferation and plasticity. Tan et al. [12] speculated that the observed decrease in dendritic cells following hAEC administration at 5 d after injury was a direct factor contributing to the decrease in the number of T cells observed at 7 d. hAECs increase the number of white blood cells in a fetal lung injury model, but levels of proinflammatory cytokines (TNF-α, IL-1 and IL-6) are simultaneously decreased [24]. These results were consistent with the early reductions in TGF-β, PDGF-α and PDGF-β levels but the increased numbers of CD45+ cells observed by Vosdoganes et al. [10] Based on these results, hAECs recruit macrophages and T cells and other inflammatory cells to the lungs during the acute stage of bleomycin-induced lung injury and then adjust the cell types to reduce inflammatory signaling; this signaling depends on the immune environment of the lung [25].

### Cytokine regulation

TGF-β, TNF-α and IL-6 play significant roles in the progression of fibrosis by mediating the epithelial to mesenchymal transition [26]. Furthermore, TGF-β is a profibrogenic cytokine, driving myofibroblast differentiation [27] and leading to collagen deposition. IL-6 and TNF-α maintain inflammation and fibrosis [28]. IL-1 is primarily secreted by monocytes and macrophages and its levels are elevated in bleomycin-injured lung tissue, thus increasing fibroblast proliferation and collagen production [29]. Levels of the TGF-β, TNF-α, IL-6 and IL-1 mRNAs were all decreased by hAEC therapy in the studies examined, suggesting that one mechanism by which hAECs mediate their effects is likely the direct regulation of the abovementioned proinflammatory cytokine levels and signaling.

The polarity of Th1 and Th2 cells is a stochastic process that is usually directed by proinflammatory (IL-6, IL-2 and IL-12) and anti-inflammatory (IL-10 and IL-4) cytokines, which provide positive feedback signaling for the downstream inflammatory response. CD11c+ dendritic cells and macrophages effectively produce IL-10 [30,31]. IL-10 levels were transiently reduced by hAECs 5 d after bleomycin injury, which was partially attributed to the reduced infiltration of dendritic cells and macrophages at 3 d [12]. Toll-like receptor-4 promotes the release of IL-6, IL-1, TNF-α and other inflammatory factors via MIF, and MIF promotes inflammation and opposes anti-inflammatory signals by regulating the MAPK pathway [32]. MIP expression was decreased by hAEC administration at 7 d after injury, confirming the anti-inflammatory effects of hAECs [13]. However, levels of the IL-10 and MIF mRNA increased at 14 d, indicating the enhanced function of pulmonary macrophages [5]. Thus, the anti-inflammatory effects of hAECs may be superior to their anti-fibrotic effects. IL-4 has been reported to be a profibrotic cytokine in preclinical studies using bleomycin. IL-4-/- mice express lower levels of profibrotic factors, including OH-proline, soluble collagen, fibronectin and TGF-β1, starting on day 21 after bleomycin injury. IL-4 limits the early recruitment of T lymphocytes and the chronic stimulation of fibrosis in a model of bleomycin-induced pulmonary fibrosis [33]. IL-4 levels were elevated by hAECs at 3 d after injury in the study by Tan et al. [12], and the authors concluded that their data illustrated the deleterious effects of hAECs on fibrotic environments and their beneficial effects under inflammatory conditions. However, we were unable to draw an accurate conclusion based on changes in levels of one factor, which should be analyzed comprehensively.

Cytokines do not exert the same effects on inflammation and fibrosis under different conditions and at different time points; researchers should consider these factors when designing clinical applications.

### hAEC engraftment in the lung

hAECs express the thyroid transcription factor, or Nkx2.1, mRNA, which is among the earliest lineage specification markers of the developing lung [34]. hAECs cultured in small airway growth medium are positive for lung epithelial markers, the production of surfactant proteins (SPs) A-D, and SP-D secretion in vitro. Low mRNA levels of IL-1, IL-10, TNF-α and TGF-β from humans have been detected in the lungs of SCID mice culled 2 weeks after hAEC transplantation, suggesting that hAECs engraft in the lung, remain viable and synthesize mRNA [5]. Additionally, differentiated hAECs retrieved from a SCID mouse model of bleomycin-induced injury at 4 weeks after transplantation produce all SPs, indicating that hAECs differentiate into type II pneumocytes in vivo. Based on these data, hAECs may be capable of longer-term alveolar restoration. Further investigations are required to determine whether the results described above will be similarly validated in immune-competent mice. Moreover, hAECs may exert preventative and reparative effects by modulating host macrophage migration to the lung, suggesting that hAEC differentiation into lung cells may not be necessary for preventing or repairing acute lung injury [10,35]. Therefore, hAECs may exert their anti-inflammatory and anti-fibrotic effects through a mechanism involving direct differentiation into alveolar epithelial cells and exert paracrine actions to regulate macrophage migration.

### Generation of suitable hAECs

hAECs must inevitably undergo in vitro amplification to generate a sufficient number of cells for treatment, leading to the epithelial to mesenchymal transformation (EMT) of hAECs and potential changes in their biological properties [36]. The P0 generation of hAECs reduces MCP-1 expression in fibrotic liver tissue [37]. The P5 generation of hAECs secrete large amounts of MCP-1 [36]. MCP-1 inhibition modulates the recruitment of immune cells into the lung tissue and plays an anti-inflammatory role. This finding may partially explain why P0 hAECs exhibit better efficacy than P5 hAECs in decreasing inflammation and modulating CD45+ cells, suggesting that original hAECs may more suitable for the treatment of lung disease [14]. In addition, the establishment of immortalized hAECs provides new opportunities for their clinical applications [38].

### Transplantation route

Different transplantation pathways are directly related to the location of hAECs in the systemic circulation in mice. Pulmonary embolism is the greatest risk of hAEC transplantation via the tail vein. I.P. hAEC injections are the main route of choice in this review and in most other stem cell studies involving lung injury animal models [3]. According to Cargnoni et al. [39], the delivery route (i.v., intratracheal or i.p.) of placenta-derived cells did not affect the engraftment rate. With a mixed population of mesenchymal and epithelial cells, the detected cells may not be hAECs, but rather mesenchymal cells. Therefore, a comparative experiment of the transplantation of hAECs alone into immunosuppressed animals via different routes should be conducted, which may provide effective guidance for the selection of cell transplantation pathways.

### hAEC source

Preterm hAECs are isolated in greater numbers and have greater proliferative capacity. However, compared to term hAECs, preterm hAECs have a more restricted differentiation potential [9]. Preterm hAECs do not express lung lineage-specific genes or surfactant protein C and D, as has been shown for term hAECs [9]. The expression of HLA-G in term hAECs is two-fold higher than in preterm hAECs. HLA-G is an antigen that decreases the proliferation of CD4+ T cells and promotes the apoptosis of CD8+ T cells, and HLA-G also adjusts the balance between Th1 and Th2 cells during pregnancy [40]. Therefore, a strategy that increases the expression of HLA-G in preterm hAECs may be beneficial to promote their anti-inflammatory effects. Term hAECs are more effective than preterm hAECs at inhibiting the migration of macrophages to the lung tissue [9]. All the aforementioned data reveal why term hAECs have been shown to decrease inflammation and fibrosis more effectively than preterm hAECs in lung fibrosis therapy [9]. Therefore, hAECs obtained from normal full-term women following Cesarean section may be more suitable for the treatment of pulmonary fibrosis. However, as shown in the study by Cargnoni et al. [39], the use of hAECs from mouse and human placenta (allogeneic and xenogeneic) does not affect their colonization in the lungs of immune-competent animals, potentially due to the detection of dead transplanted cells. Human-sourced cells have been detected by PCR or situ hybridization for human DNA or immunohistochemistry for cell markers, which are unable to clearly distinguish between dead human cells and live human cells residing in host tissues. Fluorescence-activated cell sorting (FACS) may be more suitable for detecting live transplanted cells.

### Transplantation time

An investigation of the transplantation time for hAECs in all studies examined showed that the administration of hAECs 24 h after bleomycin injury may be beneficial for inhibiting the development of inflammation in mice; thus, hAEC transplantation during the early inflammatory phase of IPF may be the most effective treatment. In addition, hAECs can directly alter fibroblast proliferation and activity to repair bleomycin-induced lung injury. Notably, the reparative effects of hAECs on lung tissue density, collagen content and α-SMA expression only emerged when the cells were delivered during the fibrotic phase of injury at day 14, but not when they were administered earlier at day 7, which is the time point of peak inflammation [10], indicating that hAECs exerted therapeutic effects on the early phase but not the peak phase of inflammation in mouse models of pulmonary fibrosis. Therefore, hAECs may have roles in preventing inflammation and in tissue remodeling by reversing the progression of pulmonary fibrosis.

### Limitations and future directions

The first round of stem cell clinical research in hospitals has been published in China [41], indicating that the clinical application of stem cells have formally expanded. The regenerative treatment of pulmonary disease urgently requires a standardized regimen. The pathological changes in IPF are very complex. Simple anti-inflammatory and anti-fibrosis effects cannot achieve ideal therapeutic efficacy, and based on the findings of this review, hAECs represent a new opportunity for regenerative therapy. However, the mouse model of bleomycin-induced lung injury primarily mimics the early phase of pulmonary fibrosis, which corresponds to only acute pulmonary fibrosis in clinical trials. The full process of IPF cannot be replicated using a bleomycin-treated animal model. Moreover, mice and other rodents recover from this injury differently than humans, posing certain limitations. Thus, the bleomycin-treated mouse model is only sufficient to reflect the acute inflammatory stage mediated by cytokines and subsequent development of fibrosis observed in patients with IPF. The long-term therapeutic effects of hAECs on IPF should be considered in clinical lung regeneration studies.

Data heterogeneity was 89% and 84% for the Ashcroft score and collagen content, respectively. This heterogeneity is derived from various factors, such as the bleomycin dose, hAEC dose, cell transplantation time, mice cull time and measurement unit (Table 1). In addition, Ashcroft score heterogeneity was attributed to subjective factors, and the heterogeneity of collagen deposition was attributed to different evaluation methods. Moreover, the P values of Egger’s test of Ashcroft scores and collagen contents were 0.003 and 0.01, which indicate existing potential publication bias. Hence, additional experiments should be performed in the future to further verify the anti-fibrotic effects of hAECs.

hAECs are more readily sourced in much greater cell numbers than bone MSCs, without the need for manipulation or expansion in vitro. In contrast to MSCs derived from Wharton’s jelly s, which do not differentiate into lung epithelium in bleomycin-treated mice, transplanted hAECs differentiate into pulmonary epithelium in the bleomycin-treated mouse model. Moreover, compared with nonspecific anti-fibrotic drugs such as pirfenidone, the dual abilities of hAECs to exert anti-fibrotic effects and differentiate into alveolar cells reflect unique advantages of this cell type. These data may suggest that hAECs are a more suitable therapeutic modality for pulmonary fibrosis.

In addition, the exocytosis of hAECs is worth exploring. Exosomes between 30–120 nm in size contain specific DNAs, mRNAs, miRNAs and lncRNAs and play important roles in epigenetics. Exosomes derived from hAECs reduce skin collagen deposition and promote scarless healing [42]. Researchers should explore whether exosomes derived from hAECs from different sources and different microenvironments exert special anti-inflammatory or anti-fibrotic effects on lung injury and determine which substances in the exosomes regulate these effects to ultimately identify a cell-free therapeutic strategy to reduce pulmonary fibrosis.

## Conclusions

hAECs alleviate the progression of bleomycin-induced lung fibrosis in mice and may represent a new clinical treatment for IPF. hAECs exert anti-inflammatory and anti-fibrotic effects by modulating the levels of macrophages, neutrophils, T cells, dendritic cells and related cytokines in mouse bleomycin-induced lung fibrosis models. Furthermore, cell generation and the route, source and timing of hAEC transplantation are all factors that determine the therapeutic effectiveness of hAECs. These factors must be considered when human studies of hAECs therapy for IPF are performed in the future.

## Supporting information

S1 Table PRISMA checklist. (DOCX)

S2 Table Risk of bias assessment of the included studies. (DOCX)

S3 Table Literature search terms (used in PubMed). (DOCX)
