# Supplementary material for: Use of human amniotic epithelial cells in mouse models of bleomycin-induced lung fibrosis: A systematic review and meta-analysis
Source: PLoS One. 2018 May 17;13(5):e0197658. doi: 10.1371/journal.pone.0197658 (PMC5957433; doi:10.1371/journal.pone.0197658)
Supplement: S2 Table — (DOCX) [file pone.0197658.s002.docx]

**Supplementary Table 2.** SYRCLE Risk of Bias Assessment for included studies.

| Author (Year) | Random sequence generation? | Groups similar at baseline? | Allocation concealed? | Animals randomly housed? | Blinding of caregivers and/or examiners? | Random selection for outcome assessment? | Blinding of outcome assessor? | Incomplete outcome data addressed? | Free from selective outcome reporting? | Free from other bias? |
| --- | --- | --- | --- | --- | --- | --- | --- | --- | --- | --- |
| Moodley 2010 | Unclear | Yes | Unclear | Unclear | Unclear | Unclear | Yes | Yes | Yes | Yes |
| Moodley 2013 | Unclear | Yes | Unclear | Unclear | Unclear | Unclear | Yes | Yes | Yes | Yes |
| Murphy 2012 | Unclear | Yes | Unclear | Unclear | Unclear | Yes | Yes | Yes | Yes | Yes |
| Murphy 2011 | Unclear | Yes | Unclear | Unclear | Unclear | Yes | Yes | Yes | Yes | Yes |
| Lim 2013 | Unclear | Yes | Unclear | Unclear | Unclear | Yes | Yes | Yes | Yes | Yes |
| Vosdoganes 2013 | Unclear | Yes | Unclear | Unclear | Unclear | Yes | Yes | Yes | Yes | Yes |
| Tan 2014 | Unclear | Yes | Unclear | Unclear | Unclear | Unclear | Yes | Yes | Yes | Yes |
| Tan 2017 | Unclear | Yes | Unclear | Unclear | Unclear | Unlcear | Yes | Yes | Yes | Yes |
| Zhu 2016 | Unclear | Yes | Unclear | Unclear | Unclear | Unclear | Yes | Yes | Yes | Yes |
