# Supplementary material for: Use of human amniotic epithelial cells in mouse models of bleomycin-induced lung fibrosis: A systematic review and meta-analysis
Source: PLoS One. 2018 May 17;13(5):e0197658. doi: 10.1371/journal.pone.0197658 (PMC5957433; doi:10.1371/journal.pone.0197658)
Supplement: S3 Table — (DOCX) [file pone.0197658.s003.docx]

**Supplementary Table 3. Literature Search Terms (Used in PubMed)**.

((((((((((((((((((((((((((((((((((((((Epithelial Cells) OR Cell, Epithelial) OR Cells, Epithelial) OR Epithelial Cell) OR Squamous Epithelial Cells) OR Cell, Squamous Epithelial) OR Cells, Squamous Epithelial) OR Epithelial Cell, Squamous) OR Epithelial Cells, Squamous) OR Squamous Epithelial Cell) OR Squamous Cells) OR Cell, Squamous) OR Cells, Squamous) OR Squamous Cell) OR Transitional Epithelial Cells) OR Cell, Transitional Epithelial) OR Cells, Transitional Epithelial) OR Epithelial Cell, Transitional) OR Epithelial Cells, Transitional) OR Transitional Epithelial Cell) OR Glandular Epithelial Cells) OR Cell, Glandular Epithelial) OR Cells, Glandular Epithelial) OR Epithelial Cell, Glandular) OR Epithelial Cells, Glandular) OR Glandular Epithelial Cell) OR Columnar Glandular Epithelial Cells) OR Cuboidal Glandular Epithelial Cells) OR Adenomatous Epithelial Cells) OR Adenomatous Epithelial Cell) OR Cell, Adenomatous Epithelial) OR Cells, Adenomatous Epithelial) OR Epithelial Cell, Adenomatous) OR Epithelial Cells, Adenomatous)) AND Amniotic) AND Pulmonary)) NOT ((review[Publication Type]) OR review literature as topic[MeSH Terms])
